# Numerical analyses of a reference wing for combination of hybrid laminar flow control and variable camber

**DOI:** 10.1007/s13272-022-00598-y

**Published:** 2022-07-19

**Authors:** Mauricio M. Jentys, Tim Effing, Christian Breitsamter, Eike Stumpf

**Affiliations:** 1 Chair of Aerodynamics and Fluid Mechanics, Technical University of Munich, Boltzmannstraße 15, 85748 Garching, Germany; 2 Institute of Aerospace Systems, RWTH Aachen University, Wuellnerstraße 7, 52062 Aachen, Germany

**Keywords:** Hybrid laminar flow control, Variable camber, Drag reduction

## Abstract

The objective of the LuFo VI-1 project CATeW (Coupled Aerodynamic Technologies for Aircraft Wings) consists in multifidelity analyses to assess the potential for aerodynamic efficiency increase by combined application of hybrid laminar flow control and variable camber technologies to the wing of a transonic transport aircraft. Individually, both technologies have proven to lead to major aerodynamic drag reductions. An evaluation of the coupled technologies is, therefore, expected to show an even higher potential due to synergy effects. To derive conclusions on system level, low-fidelity (LowFi) overall aircraft design methodologies will be applied for the analysis of a medium haul reference aircraft in the course of the project, while complex aerodynamic phenomena are modelled with high-fidelity (HiFi) computational fluid dynamics methods. The paper at hand presents results of aerodynamic analyses on both fidelity levels for the wing of the turbulent reference configuration CATeW-01, featuring the technology combination as a retrofit. Furthermore, this work encompasses adaptations and implementations performed within both the LowFi and HiFi toolchains. The LowFi toolchain already incorporates several modules for the proposed technology combination. A short presentation of the LowFi-toolchain is given, along with the modeling approach in the HiFi framework using mesh deformations and a mass flux boundary condition. Comparative studies of the turbulent flow field around the wing show good agreement of predicted load distributions in both numerical frameworks, studies based upon the HiFi approach attest the potential for efficiency increase due to the variable camber technology, incorporated by means of Adaptive Dropped Hinge Flap (ADHF) deflections. Considering the coupled application, four different constant suction mass flow rates are examined, where the maximum mass flow causes laminar flow extending over the entire suction panel, thus moving the transition location from the wing’s leading edge to the end of the suction panel. When being coupled with ADHF deflections, again the variable camber technology leads to a reduction of the wing’s pressure drag component with the simultaneous application of boundary layer suction further promoting drag reduction with increasing suction rate. While the combined application shows no mutual inhibition, major reciprocal effects are not directly observable when applying the combination as a retrofit to the reference configuration CATeW-01. This is mainly attributed to the limited extend of laminar flow, thus indicating the necessity for optimization in wing geometry and operating parameters, to achieve extensive areas of laminar flow and to promote the aspired synergy effects.

## Introduction

For conventional passenger aircraft design, a major focus is on fuel efficiency and the associated reduction of emissions. Especially in times of COVID-19, increasing aircraft efficiency is a key element to overcome the associated crisis in the aviation industry [1]. While in the past, the main priority was on revealing potential of new innovative technologies, the next reasonable step is to combine promising ideas. Two promising technologies increasing aerodynamic efficiency are variable camber (VC) and hybrid laminar flow control (HLFC). The positive effects of the individual technologies on both aerodynamics and overall performance of an aircraft have already been intensively researched, for instance considering VC by Szodruch [2] or Bolokin et. al [3] and HLFC by Braslow [4], Joslin [5] and Schrauf et al. [6]. Their coupling, however, is still an open research topic and, therefore, being addressed in the German LuFo VI-1 project *CATeW* (Coupled Aerodynamic Technologies for Aircraft Wings).

In CATeW, both technologies are applied simultaneously on the wing of a medium-haul reference aircraft on preliminary aircraft design level. The main objective of the combination consists in aerodynamic drag reduction for design and off-design cruise flight conditions due to synergy effects when combining both individual technologies. This shall be achieved by assuring an extensive laminar boundary layer through HLFC and promoting the latter through favourable adaptation of the wing loading by means of VC technologies for the entire cruise flight envelope. For consistent evaluation on overall aircraft design (OAD) level, using an interface like the “Multidisciplinary Integrated Conceptual Aircraft Design and Optimization environment” (MICADO) developed at the Institute of Aerospace Systems (ILR) of RWTH Aachen University, is inevitable. To ensure high aerodynamic accuracy, complementary High-Fidelity (HiFi) computational fluid dynamics (CFD) analyses with the DLR TAU Code [7] are performed to reflect complex, three-dimensional flow phenomena, which ultimately shall be coupled with Low-Fidelity (LowFi) methods by applying Reduced Order Models (ROMs) to MICADO.

In this work, the authors present HiFi results for the coupling of the previously mentioned technologies as a retrofit to the wing of a mid-range transonic transport aircraft. Furthermore, the applied numerical modeling techniques as well as current challenges within CATeW are discussed within this paper. Therefore, the work is divided into the following parts:

- Introduction of the reference geometry used for this work (Sect. 3);
- Overview of the approaches used in CATeW for both LowFi and HiFi (Sect. 4);
- Presentation of current challenges and HiFi results for the reference configuration CATeW-01 (Sect. 5).

The following section provides a brief overview of the relevant basics.

## Fundamentals and state of the art

The reference aircraft configuration used in CATeW is based on the OAD version of the AVACON Research Baseline 2028 (ARB2028) derived from the German LuFo project AVACON [8]. The backward swept wing of this reference requires consideration of different instability mechanisms when it comes to boundary layer transition at the wing surface. The most critical phenomena leading to transition from laminar to turbulent flow are Tollmien–Schlichting instabilities (TSI), cross-flow instabilities (CFI), and the attachment-line transition (ALT). Whereas positive (adverse) pressure gradients downstream of the maximum thickness of an airfoil amplify the two-dimensional Tollmien–Schlichting waves, the three-dimensional cross-flow instabilities predominate in regions close to the leading edge of a swept wing. This is due to the additional pressure gradient along the wingspan with increasing leading edge sweep and the accompanying three-dimensionality of the boundary layer [9]. Additionally, for swept wings, ALT can cause fully turbulent flow over the surface due to propagating disturbances along the attachment line of the flow [10].

A promising technique to suppress all instability mechanisms and, therefore, delay transition is hybrid laminar flow control: suction up to the front spar and simultaneous shaping of the pressure distribution along the airfoil in the mid-region combines the benefits of both active laminar flow control and passive natural laminar flow. Current research focuses on the potential of simplified suction concepts introduced in the early 2000s [11]; flight tests recently demonstrated the potential of these concepts on the vertical tailplane of an Airbus A320 [6].

In the context of OAD, decreasing aircraft weight by burning fuel results in changing lift coefficients for steady level flight during a mission. Since an aircraft is mostly optimized for its design point and thus for one specific lift coefficient, this inevitably leads to penalties in performance. For fixed wing geometries, a continuous climb during cruise would be the most efficient approach to counteract this. Due to lower air density with increasing altitude, this would allow to remain in the aerodynamic optimum over the whole cruise segment. However, such a procedure is not possible due to restrictions from air traffic control. Therefore, the only way to return to the optimum lift coefficient is to perform step climbs; this results in the typical sawtooth-like contour of the lift coefficient profile. Nonetheless, it also implies the aircraft eventually flying most of its mission in an off-design point. [12] The general idea of the VC technology is to minimize this deficit by adapting the airfoil geometry during flight and thereby adjusting the optimum lift-to-drag ratio of the wing to the currently required lift coefficient and mission parameters [2].

In general, there are two different categories of VC on airfoil level, namely adaption of the whole airfoil or adaption of single regions or components of the airfoil. Recent examples of the first category, e.g. the Variable Camber Compliant Wing [13], promise great potential using compliant mechanisms and no discrete control surfaces. Nonetheless, for the combination of HLFC and VC, the use of Krueger flaps at the leading edge is currently the go-to solution to prevent contamination of the surface and thus premature boundary layer transition while ensuring sufficient high-lift performance [14, 15]. This limits the feasible VC concepts to the second category and more precisely to the trailing edge of the wing; promising examples are, e.g., the Adaptive Dropped Hinge Flap (ADHF), as already used on the family of Airbus A350 [16, 17] aircraft or the Variable Camber Continuous Trailing Edge Flap (VCCTEF), developed by NASA [18, 19].

For the combination of HLFC and VC, there is already research suggesting that VC can maintain specific pressure distributions that are beneficial for HLFC (see, e.g. Greff [20] or Edi et al. [21]). Whereas past research concentrated on the aerodynamic interactions between these two technologies, CATeW broadens the matter by aiming for evaluation of the impact on the overall aircraft and the identification of dependencies between different design parameters. Before presenting first aerodynamic results that will serve as the basis of future ROMs, the reference wing is introduced and both LowFi and HiFi aerodynamic computational methods used in CATeW are discussed.

## Geometry

The aircraft reference configuration CATeW-01 is set up using the OAD tool chain MICADO [22, 23], under the scope of requirements for a mid-range mission encompassing fully turbulent flow. The wing of the configuration is presented in Sect. 3.1, along with key geometrical and mission related figures. The wing’s VC capability is implemented using ADHF deflections, which is discussed in more detail in Sect. 3.2.

### Reference wing CATeW-01

**Fig. 1:**
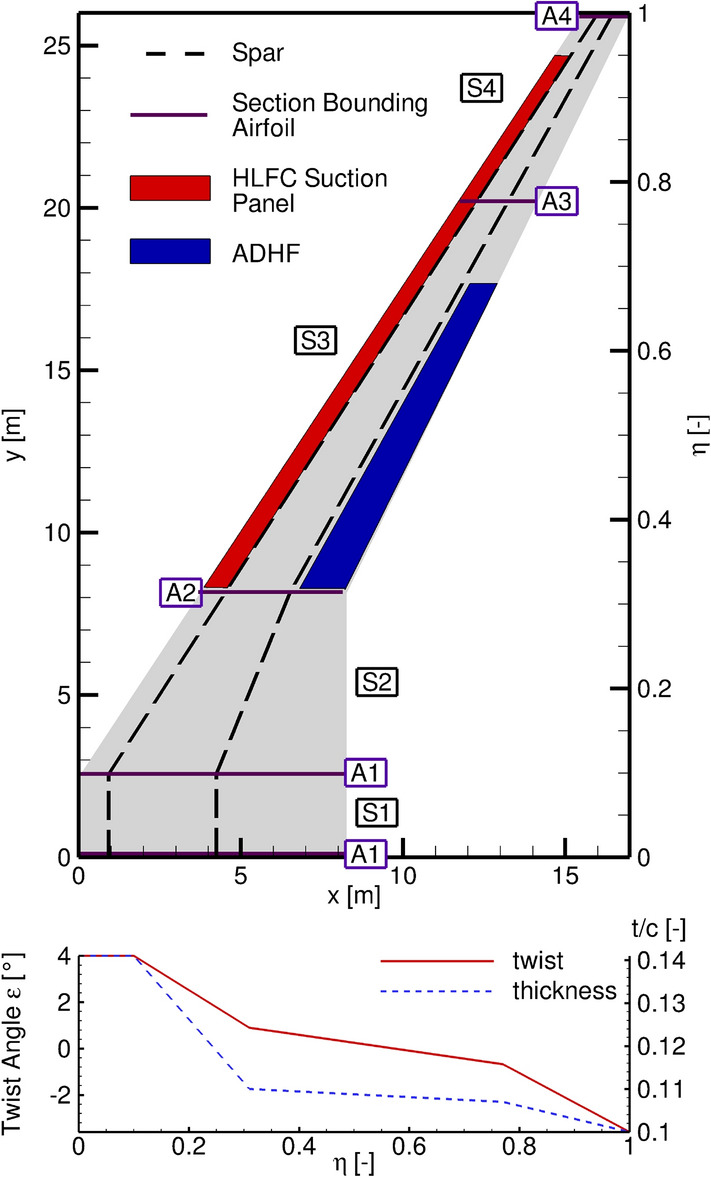
Planform of the reference wing CATeW-01. The wing consists of four sections (S1–S4), limited by adapted CRM airfoils (A1–A4, purple). The HLFC suction panel is marked in red and extends from $\eta = 0.32-0.95$ in spanwise and from the wing’s leading edge up to the front spar (dashed line) in chordwise direction. Additionally, the spanwise twist $\epsilon$ and thickness ratio *t*/*c* is plotted

As stated in the beginning of Sect. 2, a given reference aircraft is used for the CATeW project. To allow for an assessment of the technology combination in a retrofit context, the studies conducted within this paper focus solely on the main wing of this reference configuration. As indicated above, the reference configuration is designed within the software tool chain MICADO [22, 23] at the ILR (RWTH Aachen University). The resulting geometry is stored and communicated in a MICADO specific Aircraft Exchange (AiX) XML file. Key mission and geometrical quantities are listed in Table 1, a planform view of the wing and characteristic three-dimensional shape parameter distributions are shown in Fig. 1.

**Table 1:** Main mission and geometry specific parameters of the reference configuration CATeW-01

Mission parameters		
Design range $\mathrm {R}_\mathrm {D}$	4600	[NM]
Cruise Mach number $\mathrm {Ma}_\mathrm {cr}$	0.83	[-]
Initial cruise altitude $\mathrm {ICA}$	35000	[ft]
Cruise $C_L$	0.5 ± 0.05	[-]

Geometrical parameters		
Wing reference area $S_\mathrm {ref}$	220.2	[m$^2$]
Wing span *b*	52	[m]
Mean aerodynamic chord $c_\mathrm {ref}$	5.29	[m]
Aspect ratio $\Lambda$	12.28	[-]
LE sweep $\varphi _\mathrm {LE}$	33.43	[$^\circ$]
TE sweep $\varphi _\mathrm {TE}$	26.01	[$^\circ$]

The wing consists of four sections (S1–S4), where the limits of each section are defined by adapted airfoils from NASA’s Common Research Model [24], see A1–A4 Fig. 1. The inboard wing comprises sections S1 and S2, where the limit to the outboard wing is characterized by the Yehudi break at $\eta = 0.31$. To derive a three-dimensional geometry and the corresponding section surfaces, the section bounding airfoils A1–A4 are connected through linear lofts. The HLFC suction panel is placed in the outboard wing section (S3 and S4) and is marked in red (Fig. 1). The suction panel extends from ${\eta } = 0.32-0.95$ in spanwise and from the wing’s leading edge at $x/c_\mathrm {loc} = 0$ up to the local frontspar position in streamwise direction, i.e. $x/c_\mathrm {loc} = 0.16$ for the inboard and $x/c_\mathrm {loc} = 0.3$ for the outboard station of the suction panel. These dimensions are chosen in compliance with HLFC-retrofit investigations conducted by Effing et al. [25].

### Variable camber integration

**Fig. 2:**
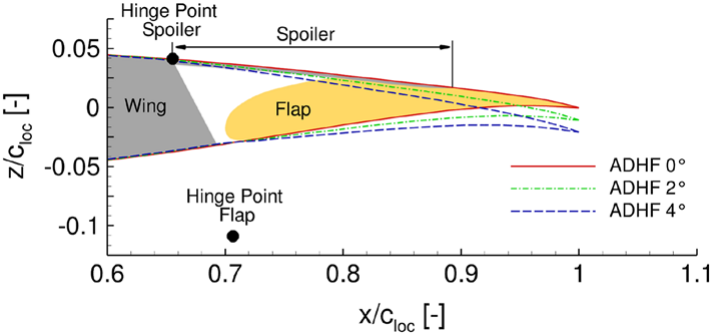
Resulting ADHF airfoil rear sections for three different deployment angles at $\eta = 0.68$. Additionally, the different components of the ADHF system are sketched with the corresponding hinge point locations for spoiler and flap rotation at $\eta = 0.68$

In the course of the project work, different integration concepts for the realization of a variable camber wing have been assessed. From the available concepts for variable camber integration, the Adaptive Dropped Hinge Flap (ADHF, see Sect. 2) has been chosen through a cost-utility analysis and accompanying two-dimensional numerical investigations, as well as expert interviews considering the technical feasibility in near future. The ADHF mainly fulfills the role of a high-lift system, nevertheless requirements during design of the latter also focused on a multi functional character of the high-lift device for usage as an enabler for a variable camber wing. The ADHF shows a rather simple deflection kinematic, namely rotation of the flap around a single hinge point placed below the wing’s trailing edge, accompanied by a downward droop of the partially overlapping spoiler. A schematic diagram of the ADHF system with its corresponding hinge points for $\eta = 0.68$ is depicted in Fig. 2.

As for the wing geometry, ADHF and spoiler specific geometry is stored in the AiX-geometry file, while the hinge point positions are transferred from known positions at an Airbus A350 wing onto the reference wing CATeW-01 using local chordlengths. The ADHF system is positioned in section S3 of the reference wing and defined through geometrical specification at the inboard and outboard stations of the flap, as summarized in Table 2. The corresponding geometrical boundary conditions at intermediate spanwise stations are linearly interpolated, allowing for construction of a three-dimensional wing geometry with extended flaps following the lofting procedure introduced in Sect. 3.1.

**Table 2:** Geometrical quantities for the ADHF of the CATeW-01 configuration. The quantities define the inboard (IB) and outboard (OB) sections of the ADHF flap

	IB	OB
Flap limit positions $\eta _\mathrm {F}$	0.31	0.68
Relative chordlength ${c_\mathrm {ADHF}}/{c_\mathrm {loc}}$	0.3	0.3
Relative hinge position $x_\mathrm {H}/c_\mathrm {loc}$	0.747	0.708
Relative hinge position $z_\mathrm {H}/c_\mathrm {loc}$	− 0.097	− 0.112

## Computational methods

The aerodynamics of the reference wing CATeW-01 are modeled with numerical techniques of different fidelity levels, which are presented in the following sections. Section 4.1 elaborates on the aerodynamics module of MICADO, representative of the LowFi Methods. Subsequently, the applied CFD methods within the framework of the DLR TAU Code are described in Sect. 4.2, forming the corresponding HiFi complement.

### LowFi: aerodynamic module in MICADO

In contrast to high-fidelity CFD simulations, the common approach in OAD is to determine the different drag components independently and then calculate the total drag as the sum of the single components. The basic approach in the aerodynamic module in MICADO, therefore, combines the multi-lifting-line code LIFTING$\_$LINE (LILI)^1^ for the induced drag and well-known semi-empirical relations for the other components, such as viscous and wave drag. To increase the level of detail for HLFC applications, Risse [29] developed a so-called 2.5D method to replace the semi-empirical relations by Raymer [30] and Korn-Mason [31, 32] used for the wing’s viscous and wave drag, respectively. In the following, a short but not exhaustive overview of the approach is given; for detailed information the reader is referred to Risse [29].

The basis for the method are geometric and fluid-mechanics transformation rules which allow the iterative coupling of the 2D Euler/Boundary layer flow solver MSES [33] with the 3D stability analysis program suite STABTOOL [34, 35]; the latter enables the consideration of both TSI and CFI and subsequently the prediction of the transition location via the $e^N$ method and the $N_{CF}$-$N_{TS}$ method, respectively [36]. In addition, ALT is evaluated using the Pfenninger–Poll criterion that was derived from ONERA wind-tunnel tests [37].

To allow for fast and robust drag prediction within the MICADO loop, the 2.5D process chain is used a-priori to set up a database for given airfoil geometries along the wing span. These geometric key points are primarily the inner and outer boundaries of the known wing geometry. For each geometric key point, both turbulent and laminar aerodynamic data for numerous variations of Mach number and lift coefficient are calculated and subsequently written in the database to provide data for the whole flight envelope.

Within the MICADO loop, the aerodynamic module starts with calculating local lift distributions and induced drag for a predefined number of angles of attack using LILI. For the wing drag, the database is then queried for various spanwise wing positions and the respective local lift coefficients. Whenever a queried position is between two geometric key points, the corresponding data is interpolated. Thus, a single key point influences not only its specific spanwise position, but also the entire segment. For example, the aerodynamic properties of S2 in Fig. 1 depend on the airfoil characteristics of both the root airfoil A1 at $\eta =0.1$ and the kink airfoil A2 at $\eta =0.31$. As a last step, the total wing drag is derived from the sum of the area weighted section drag components.

Although this approach has some outstanding benefits, especially the transformation rules applied for geometry, freestream conditions, and pressure distributions underlie some uncertainties. This was already highlighted by Effing et al. [25]; the future handling of these uncertainties is one of the objectives of CATeW.

The VC technology comes in place by setting up a database not only for the clean airfoils of each geometric key point but also for various permutations of the eligible airfoils. The general idea is to calculate drag polars for every possible combination of airfoil permutations along the wingspan and subsequently merge these according to a predefined criterion, such as the best lift-to-drag ratio [38]. The permutations are realized using an in-house tool, which creates ADHF airfoils based on predefined geometrical quantities for the ADHF flaps (see Table 2; additionally, the tracking of the partially overlapping spoiler is also considered). The deployed geometry finally used in the 2.5D approach results from the envelope of the single components of the ADHF airfoil slice. Fig. 2 exemplifies both the initial clean airfoil with the individual components and the resulting ADHF airfoils with different deployment angles.

### HiFi: computational fluid dynamics

For CFD simulations, the DLR TAU Code [7] is applied. The TAU Code is an unstructured, three dimensional finite volume solver using a vertex-centered dual cell scheme. The compressible RANS equations are solved in the present case, on one hand fully turbulent using the $k-\omega$ SST [39] turbulence model and on the other hand incorporating transition using the correlation based $\gamma$-${Re}_\theta$ model. The $\gamma$-${Re}_\theta$ model builds upon the $k-\omega$ SST model by solving two additional transport equations, one for the intermittency $\gamma$ and one for the local transition onset momentum thickness Reynolds number $\widetilde{{Re}}_{\theta ,t}$, which links semi-empirical correlations with respect to boundary layer transition with the boundary layer’s intermittency $\gamma$. The latter controls the production and destruction terms in the model’s turbulent kinetic energy transport equation. Values of $\gamma = 1$ correspond to a fully turbulent and values of $\gamma = 0$ to a fully laminar cell state, for which the production and destruction terms of turbulent kinetic energy in the respective cells are limited. [40]

Since the original model by Langtry et al. [40] is only capable of accurately predicting transition due to two-dimensional transition mechanisms, namely TSI, an extended version developed to also account for CFI by Grabe et al. [41] by means of the helicity Reynolds number $Re_{He}$ is applied.

#### Solver settings and computational grid

For spatial discretization of the RANS equations, a central scheme with scalar dissipation is applied, time stepping is performed using an implicit Backward Euler scheme. The resulting linear system of equations is solved using a lower–upper symmetric Gauss-Seidel scheme and a 3w multigrid cycle is applied for convergence acceleration.

In the linear range of the $C_L$-polar, steady state simulations are performed and a target $C_L$ algorithm for determination of the corresponding angle of attack $\alpha$ is applied. When further increasing the angle of attack, transient effects occur which are taken into account by switching to a dual-time stepping scheme with a physical time step of $t = 1\cdot 10^{-4}\text { }\mathrm {s}$, derived from the suggestion given by Frink [42].

**Fig. 3:**
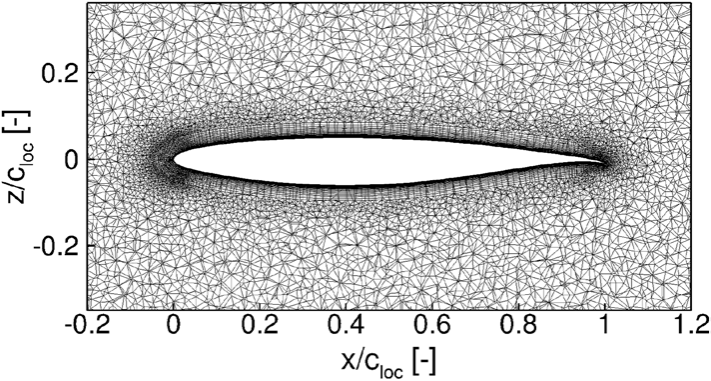
Slice through the volume mesh at $\eta = 0.5$

The computational grid was generated using the commercial, hybrid grid-generator CENTAUR by CentaurSoft. The boundary layer of the configuration lies within a prism cell stack with a growth ratio of 1.1 and a first cell height satisfying $y^+<1$. The entire computational domain forms a hemisphere with a radius of 100 reference chord lengths and is filled up by tetrahedral elements, where a refinement box is placed in the near field of the reference geometry. The general grid generation philosophy is based on the guidelines presented for the AIAA Transition Modeling and Prediction Workshop [43]. A slice through the final volume mesh at ${\eta } = 0.5$ is shown in Fig. 3.

**Fig. 4:**
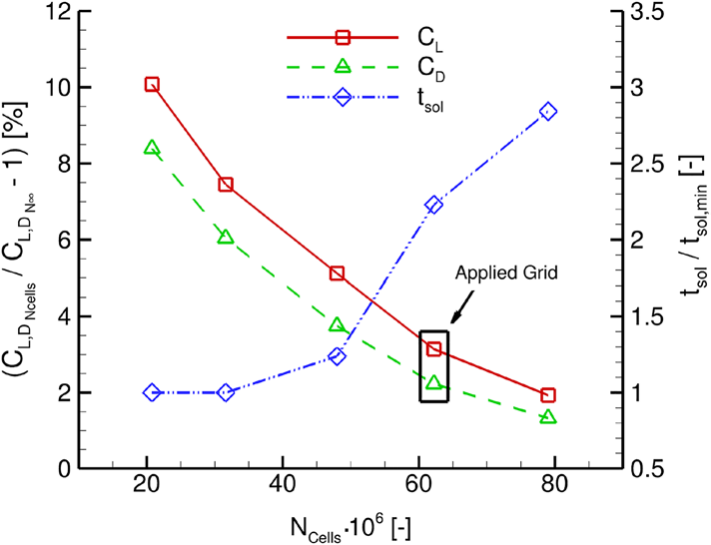
Assessment of solution convergence based on integral force coefficients following the methodology introduced by Roache in [44]

Truncation error assessment of the numerical solution with regard to grid resolution has been studied by successive refinements of the surface and refinement box discretizations with respect to pressure distributions and global force coefficients. The applied computational grid contains approximately 64$\cdot \text {10}^\text {6}$ cells, consisting of 26$\cdot \text {10}^\text {6}$ tetrahedra and 38$\cdot \text {10}^\text {6}$ prisms. Furthermore, an assessment of spatial grid convergence applying the method introduced by Roache [44] is depicted for integral force coefficients in Fig. 4.

#### Modeling of HLFC and VC

**Fig. 5:**
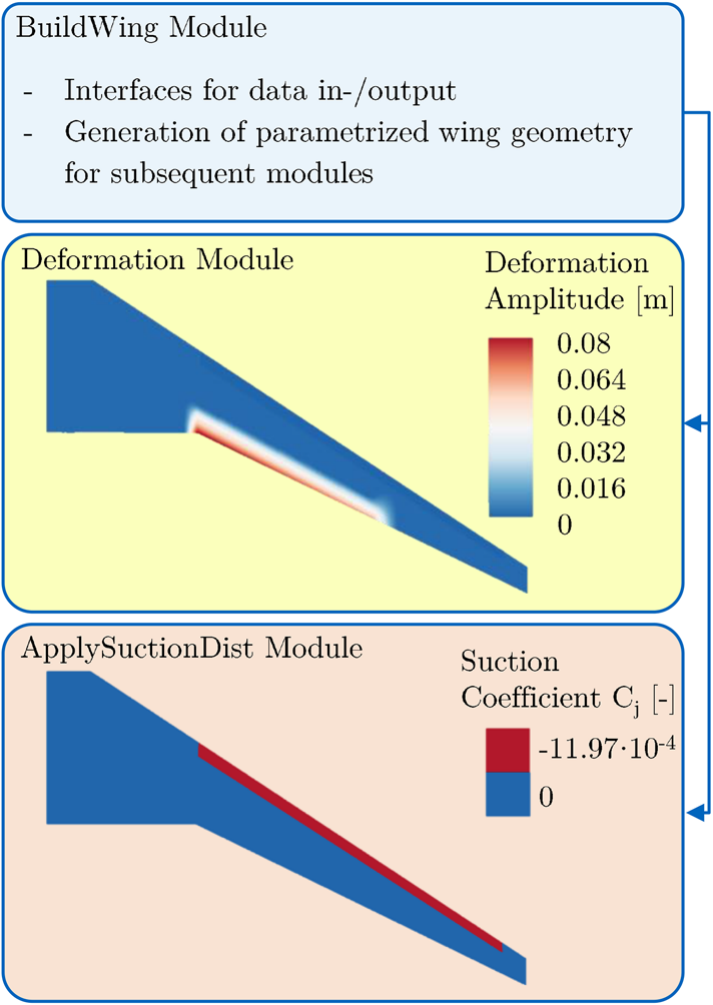
CATeW Python toolbox with different modules to model the VC-system and the HLFC suction boundary condition. The corresponding modules interact with required TAU in- and output files

A Python Toolbox for automated application of HLFC and VC specific characteristics in HiFi numerical modeling is being implemented in the course of the project CATeW. Up to this point, the toolbox consists of three main modules, where a general overview is provided in Fig. 5.

The BuildWing-Module represents the main module of the toolchain, deriving a parametrized, three-dimensional geometry consisting of sectional airfoils with corresponding surface points from the AiX-file. Based upon this geometry, deformation fields according to the ADHF kinematics and corresponding spoiler droop are computed using the deformation module. The deformation fields are subsequently passed to the TAU code’s mesh deformation module, where radial basis function interpolation is used to compute the deformed CFD grid from the scattered data files output by the Python toolbox deformation module. This modeling approach embedded in TAU’s capabilities is presented amongst others by Alcaraz Capsada et al. [45] and does not take into account spanwise gaps/discontinuities in the geometry due to flap deflections. Nevertheless, it is applied in this work due to the similar deformation character within the LowFi-toolchain and the computationally cheap and robust implementation within an automated framework.

To account for boundary layer suction, the effusion mass flux for application of the numerical boundary condition within TAU is computed in the corresponding cells of the HLFC suction panel using the ApplySuctionDistribution module. The boundary condition incorporates a non-zero wall normal velocity to a non-slip viscous wall through specification of a mass flux *j* [46]:

1 $$\begin{aligned} j = \frac{\dot{m}}{A} = \rho V_n. \end{aligned}$$

For non-dimensional representation, a suction mass flow coefficient $C_j$ is introduced by means of the freestream density $\rho _\infty$ and velocity $U_\infty$:

2 $$\begin{aligned} C_j = \frac{\rho }{\rho _\infty } \frac{V_n}{U_\infty }, \end{aligned}$$

where the velocity ratio $V_n/U_\infty$ is typically referred to as the suction coefficient $C_q$ [25].

The respective transitional turbulence model is not directly affected by the effusion mass flux boundary condition, nevertheless computations for flat plate flows incorporating boundary layer suction via this method show correct representation of the laminar flow boundary layer velocity profile when compared to an analytical solution. Furthermore, comparison to experimental transition locations for a two-dimensional test case incorporating boundary layer suction show good agreement when applying the boundary condition together with correlation-based transition turbulence models [47].

## Results

**Fig. 6:**
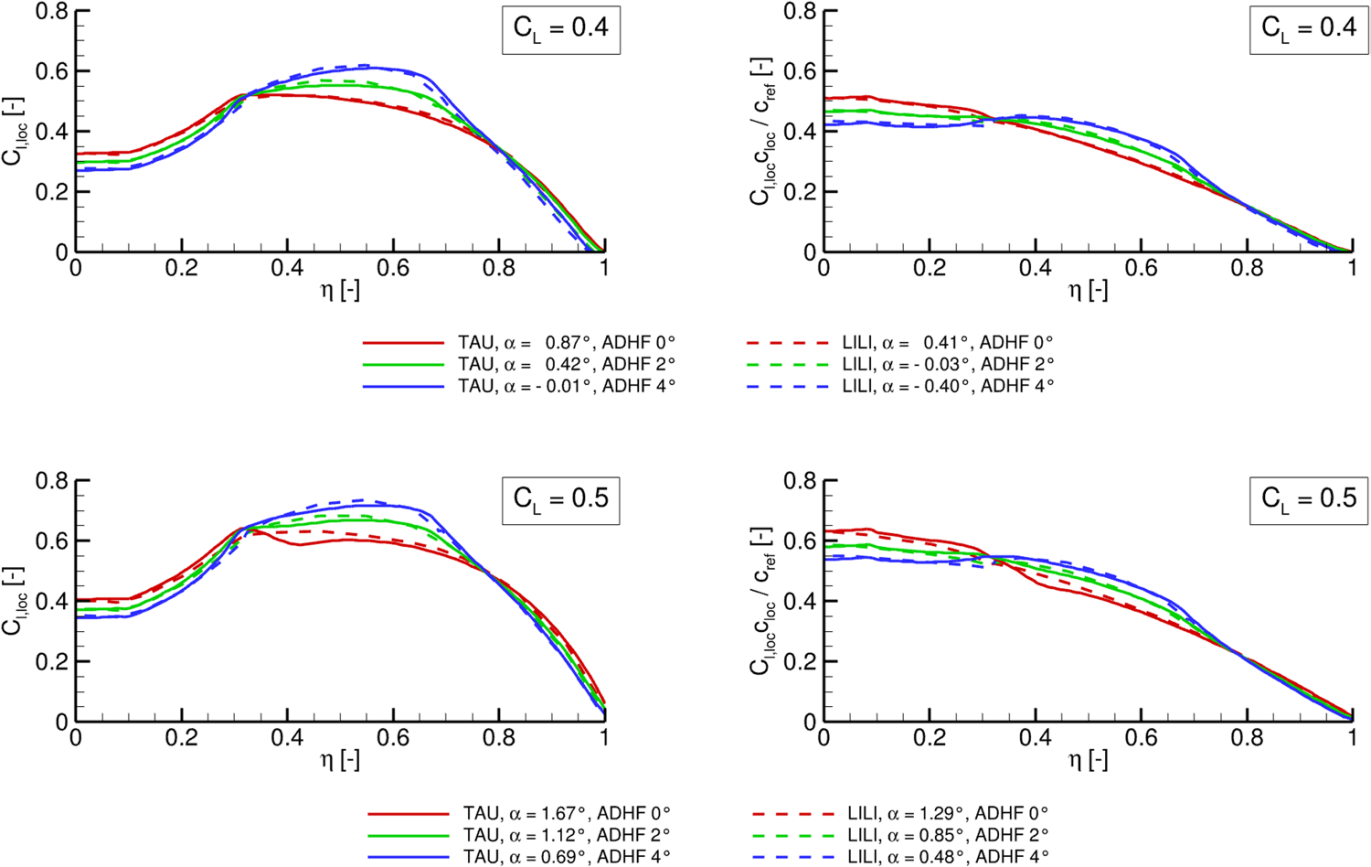
Comparison of spanwise $C_{l,loc}$ (left) and aerodynamic load distributions (right) computed with LILI and the DLR TAU code for global lift coefficients of $C_L = 0.4$ (top) and $C_L = 0.5$ (bottom)

The studies are performed at cruise Mach number ($\mathrm {Ma}_\mathrm {cr} = 0.83$) and a Reynolds number of $\mathrm {Re} = 34.2 \cdot {10}^{6}$, with respect to the reference chord length of ${c}_\mathrm {{ref}} = 5.29\text { }\mathrm {m}$ and the initial cruise altitude $\mathrm {ICA}= 35000\text { }\mathrm {ft}$. A $C_L$-$\alpha$ sweep is defined in terms of the lift coefficient $C_L$, in a range of $C_L = [0.3,0.6]$. As described above, a target $C_L$ algorithm is chosen for steady RANS computations in the linear range of the polar, whereas transient RANS computations are performed for $C_L$ values in the upper range of the sweep. ADHF deflection angles from $[-2^\circ ;4^\circ ]$ are considered, resulting in 24 ADHF-$C_L$ combinations.

Section 5.1 presents analyses considering fully turbulent flow, featuring a comparison between modeling on different fidelity levels w.r.t. aerodynamic load distributions under the effect of different ADHF deflection angles and $C_L$-polar calculations based on the HiFi-toolchain. Computations considering boundary layer transition are presented in Sect. 5.2, incorporating different constant suction mass flows and ADHF deflection angles within the HiFi-simulation framework.

### Turbulent flow

A comparison between the LowFi and HiFi computational toolchains considering the local lift coefficients $C_\mathrm {{l,loc}}$ (left) and aerodynamic loads (right) is given in Fig. 6. The distributions are shown for global lift coefficients of $C_L = 0.4$ and $C_L = 0.5$ and ADHF deflection angles of $0^\circ$, $2^\circ$, and $4^\circ$.

**Fig. 7:**
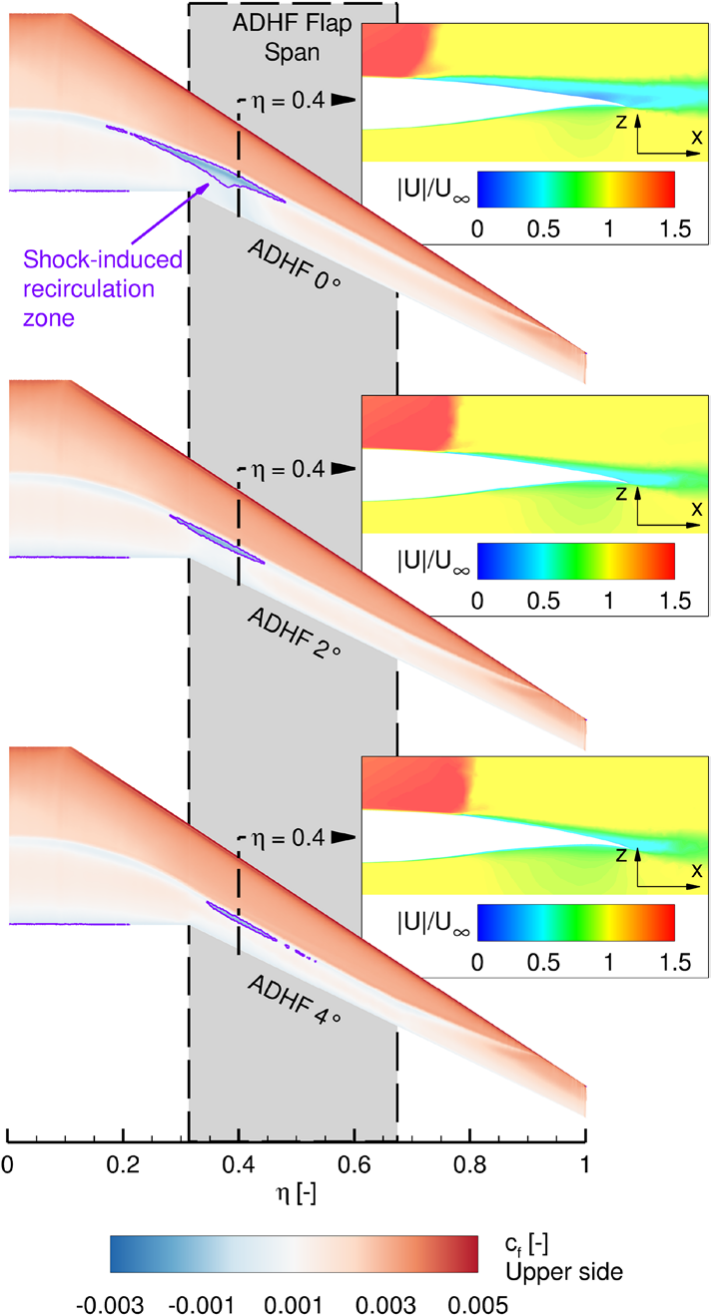
Skin friction contours for $C_L = 0.4$ and ADHF deflection angles of $0^\circ$, $2^\circ$ and $4^\circ$, along with non-dimensional contours of the flow velocity $|U|/U_\infty$ for $\eta = 0.4$ are depicted

The characteristic shapes of the distributions agree very well between the LowFi computations based on LILI results and the (U)RANS solution. The shift in $C_\mathrm {{l,loc}}$ and aerodynamic load from the inboard to the outboard wing for increasingly positive ADHF deflections is reflected in all cases. Considering the higher global $C_L$ value of $C_L = 0.5$, differences between both modeling approaches arise in the kink section of the wing at $\eta \approx 0.3$. These differences can be attributed to viscous effects in the case of the HiFi-simulations in form of a growing shock-induced separation and associated recirculation zone forming at higher angles of attack, see Fig. 7.

Based on the local lift coefficient distributions shown in Fig. 6 (left), the next step in the LowFi-toolchain consists of the extraction of corresponding sectional polars from the previously computed database, as described in Sect. 4.1. Nevertheless, this is not immediately possible for the derived geometry in the context of the 2.5D method, due to the performance of the given root airfoil not being sufficiently high. This not only affects the further possibilities for analyzing the given reference wing with the 2.5D method, but also illustrates limitations of 2.5D methods in general. Since this even increases the need for integrating ROMs into MICADO, the topic is briefly discussed next.

**Fig. 8:**
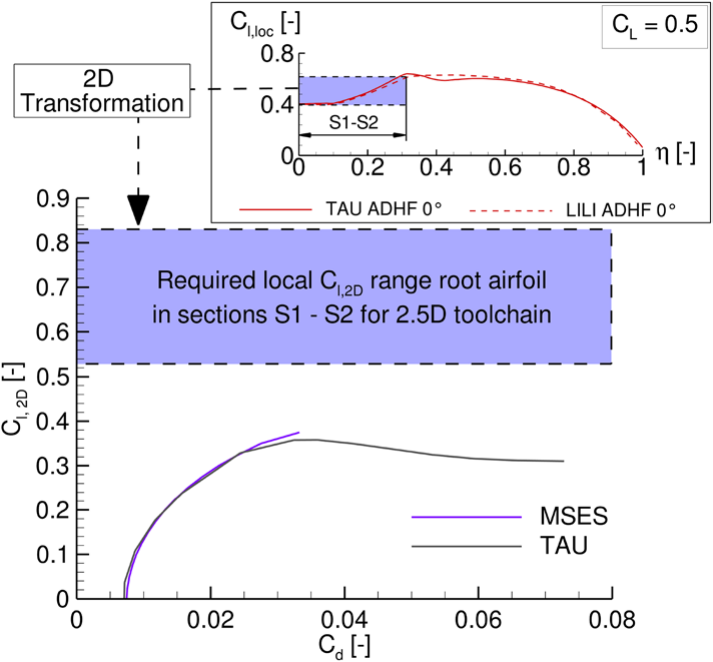
Top: $C_\mathrm {{l,loc}}$ distribution for $C_L$ = 0.5. The inboard wing S1–S2 (marked in purple) partly requires 2.5D-section polars computed from the root airfoil, which does not reach the necessary lift coefficient in 2D computations (bottom)

The current challenge associated with the root airfoil is depicted in Fig. 8, exemplarily for a global lift coefficient of $C_L=0.5$.

As already stated in Sect. 4.1, polar data of the root airfoil partly affects the drag computations for the entire inboard wing (S1–S2), characterized by local lift coefficient values of $C_\text {l,loc}>0.4$ (marked purple in the top of Fig. 8). Following the default procedure in the 2.5D method for the wing root section, these local coefficients are transformed with a reference sweep angle of a surrogate wing planform [29]. The resulting local lift coefficients $C_\text {l,2D}$ cannot be reached for the root airfoil, as shown in Fig. 8 (bottom), thus inhibiting the computation of corresponding wave and viscous drag components for sections S1–S2 in the LowFi-toolchain.

A first step to cope with this challenge is to divide the inner segment S2 into several smaller segments, thus reducing the influence of the root airfoil over segment S2. A further splitting of S1 does not provide any advantage, since the identical airfoil is used for both segment boundaries (see Fig. 1). To further counteract the insufficient performance of the root airfoil, it would also be possible to allow extrapolation of the existing data in a next step. However, since one objective of CATeW is a consistent comparison of LowFi and HiFi results to take measures based on this, extrapolation is not a preferred measure. To further exclude a major influence of the chosen solver, MSES polars are compared to 2D computations conducted with the TAU Code, showing good agreement between both solvers (Fig. 8 bottom). This discussion reveals that the challenge in this case is not the flow solver or a transformation rule (see Sect. 4.1), but rather possible limitations in the analysis of 2D airfoils: Although the wing performs well in 3D analyses, the calculation of sectional polars from 2D airfoil slices may not be possible. In the further course of the project, this might be solved by a local adaptation of the geometry and the foreseen link of LowFi and HiFi computations through the usage of ROMs based on HiFi computations.

**Fig. 9:**
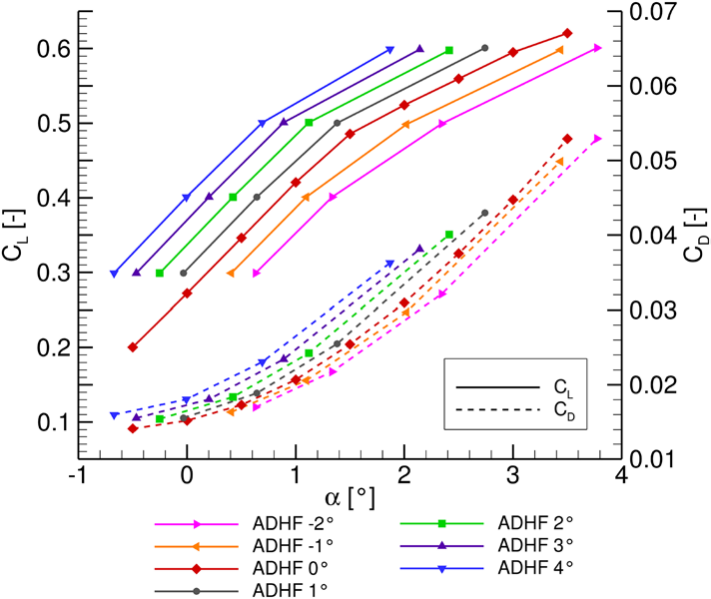
Lift $C_L$ and drag $C_D$ polars for different ADHF deflection angles

Due to the above mentioned challenges in the course of the LowFi-based computations, the following results are solely based on the HiFi CFD computations. Global lift and drag polars for the previously presented parameter range are shown in Fig. 9.

The typical behaviour connected with a camber variation at a wing’s trailing edge is reflected in the corresponding curves, namely a shift of the lift polar for constant angles of attack $\alpha$ to higher lift coefficient values $C_L$, while the maximum lift coefficient $C_{L,max}$ moves to lower angles of attack $\alpha _{max}$, where the inverted behaviour is consequently observed for the drag polar.

**Fig. 10:**
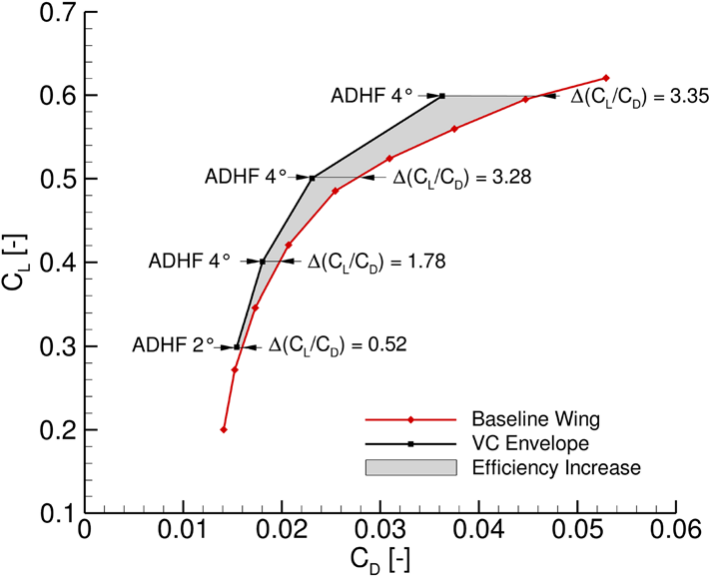
Lilienthal polar for the reference wing with ADHF 0$^\circ$ (red), the envelope of the maximum achievable lift-to-drag ratio due to ADHF deflections (black) and the corresponding efficiency increase (grey)

The combined representation of lift and drag in the form of a Lilienthal polar is given in Fig. 10. The baseline polar for an ADHF deflection angle of 0$^\circ$ is depicted in red, while the envelope of maximum efficiency increase in terms of lift-to-drag ratio for different ADHF deflection angles is shown in black.

The main part of the VC envelope is characterized by the maximum ADHF deflection angles of 4$^\circ$, an intersection of the corresponding ADHF polars takes place in the range of $C_L < 0.4$, lying outside of the design $C_L$ range. A maximum in aerodynamic efficiency increase of $\Delta (C_L/C_D)$ of 3.35 is achieved for $C_L = 0.6$ with a decrease to $\Delta (C_L/C_D) = 3.28$ for the design lift coefficient of $C_L = 0.5$. This major increase in aerodynamic efficiency can be attributed to a strong reduction of the shock-induced separation and recirculation region on the wing’s suction side for high $C_L$ values, thus reducing the maximum achievable aerodynamic efficiency increase for cases with lower global $C_L$ values.

**Fig. 11:**
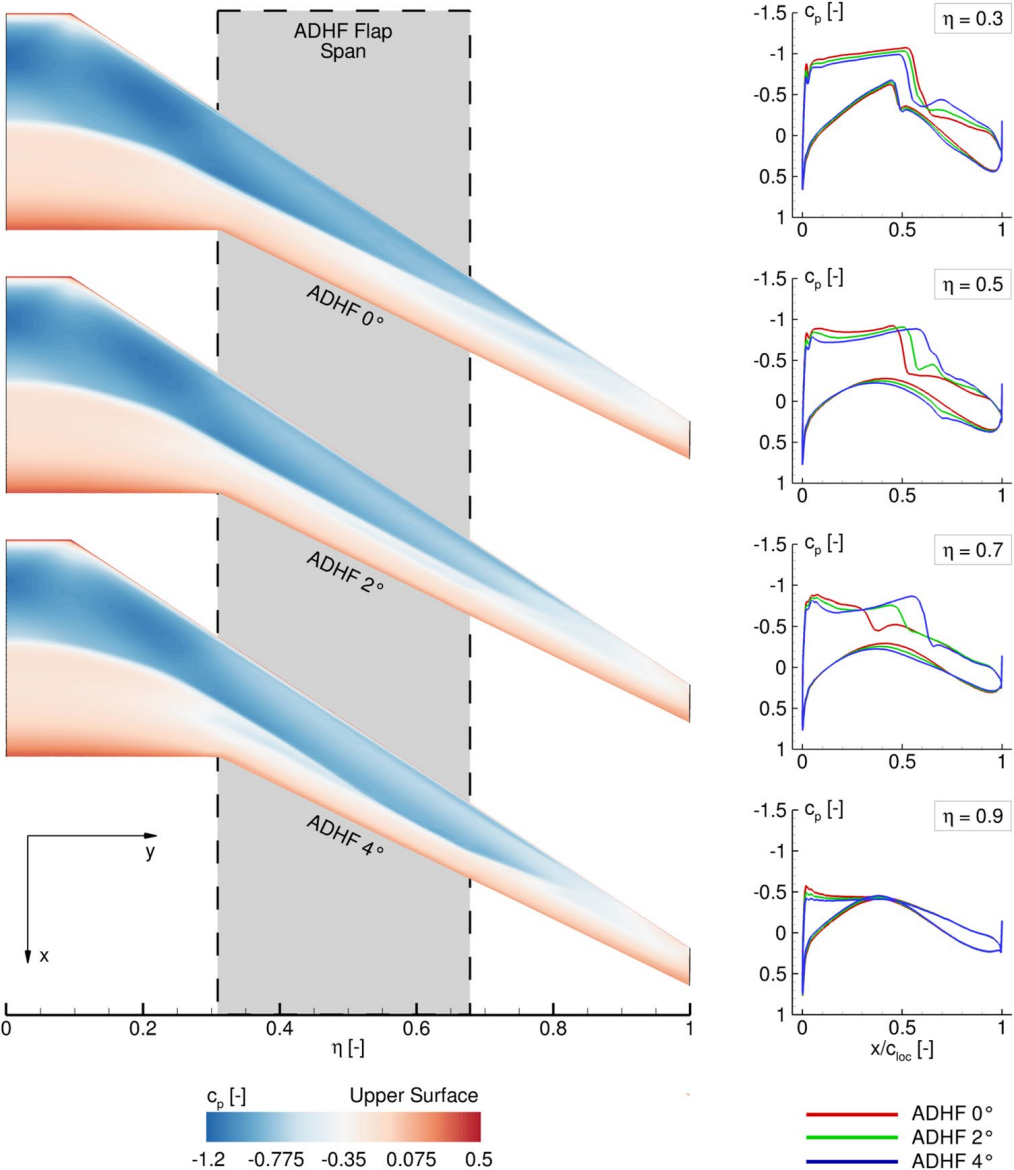
Influence of different positive ADHF deflection angles on upper surface pressure distributions (left), with additional indication of the ADHF flap extent, and extracted streamwise pressure distributions (right) for $C_L$ = 0.4

To conclude the turbulent flow analyses, the influence of selected positive ADHF deflection angles on the surface pressure distribution in terms of the dimensionless pressure coefficient $c_p$ are shown in Fig. 11 (left). While no suction is present in the turbulent cases, streamwise pressure distributions in the spanwise range of the HLFC suction panel (${\eta _{HLFC}} = 0.32-0.95$) are shown in Fig. 11 (right), to highlight the potential for active pressure distribution shaping through VC-integration in this part of the wing.

The pressure distributions are shown for $C_L = 0.4$, encompassing fully attached flow for the depicted ADHF deflection angles. Generally, the wing’s suction peak is decreased for increasing ADHF deflections. Considering the surface pressure distributions and the cuts at $\eta = 0.5$ and $\eta = 0.7$ (Fig. 11 right) the shock position is moved further downstream, enhancing the chordwise extent of negative pressure gradient flow. The shock strength is decreased considering the sectional cuts at $\eta = 0.3$ and $\eta = 0.5$, while a secondary shock associated with the contour kink due to the deflected ADHF is partially observable. Considering the pressure distribution at $\eta = 0.9$, no shock is formed due to subcritical $c_p$ levels associated with the lowest local wing twist angles $\epsilon$ in the wing tip region (see Fig. 1).

### Transitional flow

**Fig. 12:**
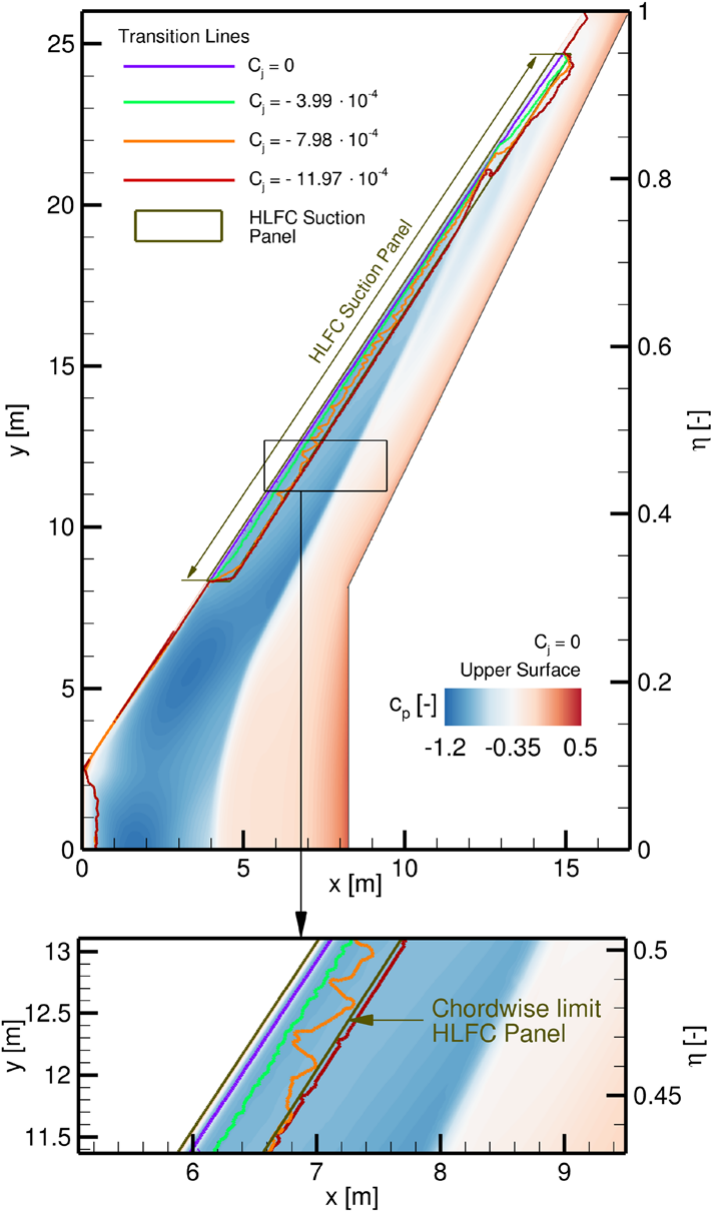
Transition line for different suction mass flows applied in the HLFC suction panel for $C_L = 0.4$ and $\text {ADHF} = 0^\circ$, with additional detail representation

As indicated above, the test matrix for the transition boundary layer analyses is equal to the turbulent analyses, while additionally four constant suction massflows with successively increasing magnitude are investigated, see Fig. 12. Furthermore, Fig. 12 shows the pressure coefficient distribution on the wing’s upper surface for $C_L = 0.4$ and zero suction as a reference, along with the transition lines for different suction mass flows.

Considering the zero suction baseline case, transition is observed in direct vicinity of the wing’s leading edge for the upper and the lower surface, respectively. This behaviour is typical for a highly swept wing in transonic regime and can be attributed to the development of strong cross-flow velocity profiles leading to transition due to CFI, see Sect. 2. A minor increase in laminar flow extent in streamwise direction is notable for increasing spanwise stations.

The investigated suction mass flow rates provide a progressively increasing laminar flow extent in streamwise direction in the marked HLFC suction panel zone, where the entire area of the suction panel is held laminar for the highest mass flow. At a span position of $\eta \approx 0.8$ the transition position is unaffected for different suction massflows due to the shock intercepting the HLFC suction panel, causing flow transition. Laminar flow exceeding the suction panel’s downstream limit is achieved outboard of the shock leading edge intersection at $\eta \approx 0.85 - 0.95$, where transition is shifted to $x/c_\mathrm {loc} \approx 0.45$ for $C_j = -11.97\cdot 10^{-4}$. Outside of the suction panel region the transition locations are not affected and match for all suction mass flows.

**Fig. 13:**
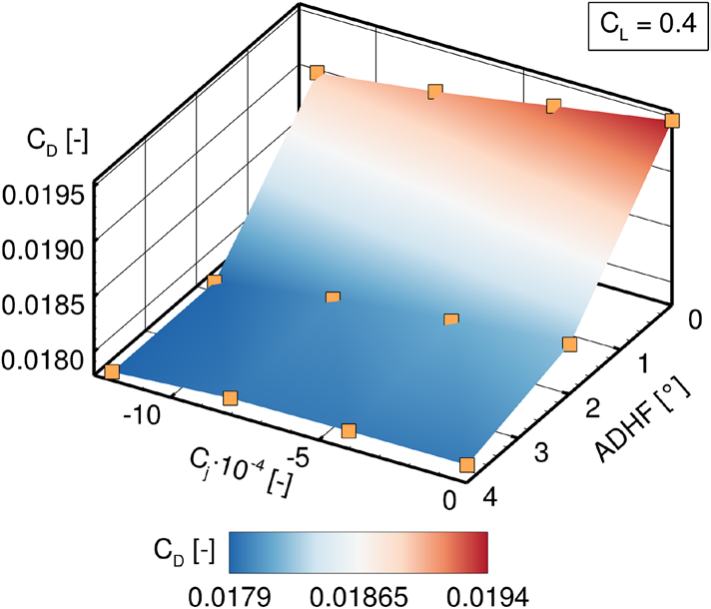
CATeW-01 drag coefficient $C_D$ for variations in both ADHF deflection angle and suction coefficient $C_j$ for $C_L = 0.4$

**Fig. 14:**
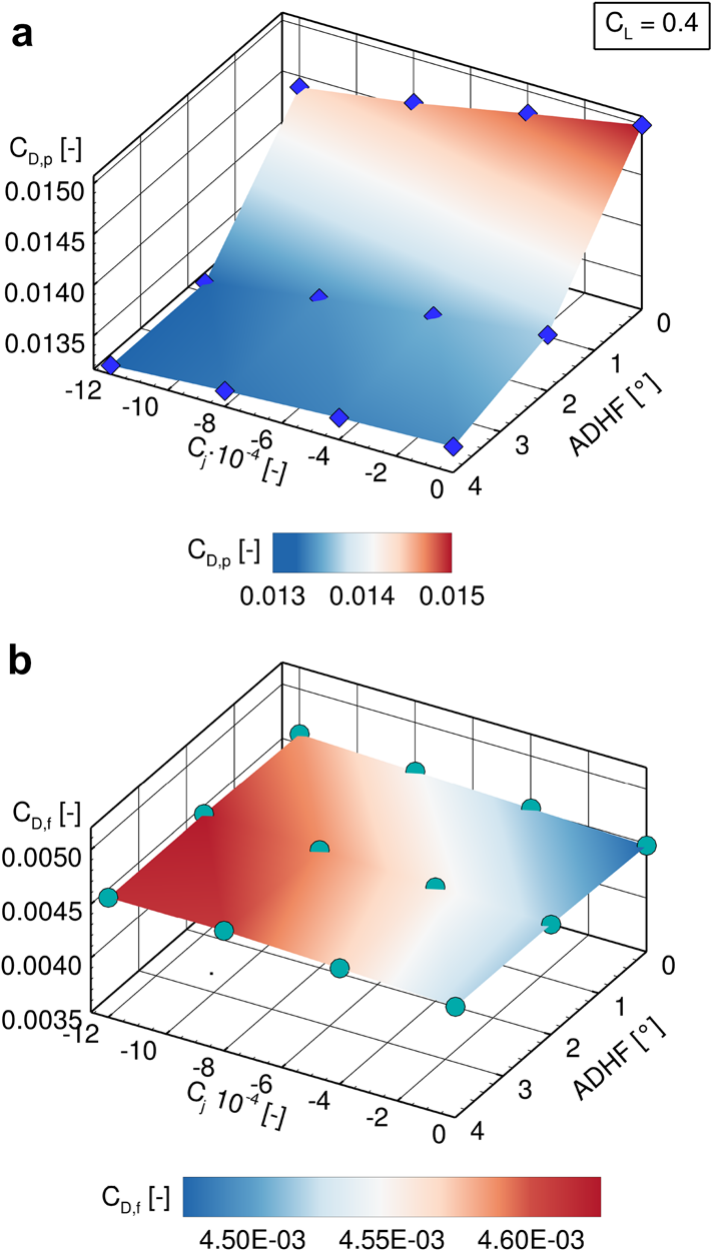
CATeW-01 drag decomposition into (**a**) pressure drag component $C_{D,p}$ and (**b**) friction drag component $C_{D,f}$ for variations in both ADHF deflection angle and suction coefficient $C_j$ for $C_L = 0.4$

Considering the coupling of VC and HLFC, the effects of parameter variations in both dimensions on the wing’s drag coefficient are shown in Fig. 13. Both an increase in ADHF deflection angle and suction coefficient lead to an overall reduction of the wing’s drag coefficient, showing the capability of both systems to be operated simultaneously with no mutual inhibition. Nevertheless, due to the limited extend of laminar flow the envisaged synergy effects cannot be fully exploited and final conclusions for the VC and HLFC coupling cannot be drawn considering the reference configuration CATeW-01, since the reduction in overall $C_D$ is attributed to a reduction in pressure drag components in both cases, see Fig. 14. Therefore, the results presented for CATeW-01 in the following reveal characteristics of a purely (active) laminar flow control (LFC) system (see Sect. 2), when being coupled to VC integration on an aircraft wing.

While the reduction in pressure drag components^2^ due to ADHF deflections has been discussed in the context of the turbulent analyses in Sect. 5.1, the reduction in pressure drag due to boundary layer suction is connected to a decrease in boundary layer thickness with increasing suction coefficient $C_j$. This effectively acts as an additional mean of camber variation, namely reducing the effect of viscous decambering [49] and thus promoting the already present VC drag reduction technique through ADHF deflection, see Fig. 14 a).

In contrast, the limited range of laminar flow achieved for CATeW-01 expresses itself in nearly constant friction drag components $C_{D,f}$ for the investigated parameter range, see Fig. 14 b). This highlights the necessity for extensive zones of laminar flow on the wing’s surface, namely through exploitation of the (passive) natural laminar flow aspect of HLFC on top of the LFC part, to achieve a marked reduction in the friction drag components and to promote the technology coupling’s synergy effects in the primarily intended way.

For further assessments, a comparison with the LowFi-Methodology needs to be undertaken, for which geometry adaptations might be necessary. Furthermore, suction in the HLFC panel considering the HiFi toolchain should be defined in terms of suction velocity or the suction coefficient $C_q$, respectively, rather than prescribing the suction mass flow. For instance the above shown comparisons (Fig. 14) do not feature the same suction velocity distributions, due to the prescribed effusion mass flow coupling the wall normal velocity to the density on the wing’s surface, which is significantly altered for different ADHF angles at constant $C_L$. Therefore, the boundary condition needs to be reassessed for the above mentioned requirement, either directly in the solver’s source or by implementation of a non-intrusive matching algorithm for prescribed velocity profiles, avoiding the necessity of altering the underlying boundary condition.

## Conclusions and outlook

This paper aims at presenting the LuFo VI-1 project CATeW (Coupled Aerodynamic Technologies for Aircraft Wings), where the potential for efficiency increase due to a coupling of hybrid laminar flow control (HLFC) and variable camber (VC) technology to a transonic transport aircraft wing should be assessed. It lays the foundation for the analyses, presenting the assessment of the technology coupling in a retrofit context to the wing of a turbulent reference configuration CATeW-01 on two different fidelity levels. The LowFi framework is presented by MICADO and HiFi analyses are performed using the DLR TAU Code, alongside specifically implemented extensions implemented for the project to model the technology coupling. Furthermore, the comparisons between LowFi- and HiFi-frameworks presented within this work are prerequisites for the project’s dedicated goal of including HiFi aerodynamic results into the LowFi-framework via reduced order models. This will allow for a synergistic use of HiFi fluid dynamics results in overall aircraft design for a technology assessment on system level.

The analyses and comparisons between both solver frameworks agree well with respect to load distributions and two-dimensional analyses. Since the deducted geometry faces substantial challenges with respect to viscous calculations within the LowFi-toolchain, further assessments of the turbulent reference configuration CATeW-01 are conducted based on HiFi-analyses, showing the VC potential for drag reduction encompassing fully turbulent flow.

Computations with the four equation $\gamma$-${Re}_\theta$ model show transition being predicted in direct vicinity of the wing’s leading edge. A downstream shift in transition location is achieved with increasing constant suction mass flow, while laminar flow extends maximally to the end of the HLFC suction panel for the present study, for which only the HLFC system’s (active) laminar flow control (LFC) component can be considered as a basis for conclusions for CATeW-01. Both an increase in ADHF deflection angle and an increase in suction strength lead to an overall drag reduction for constant lift coefficients. While the ADHF deflection’s working on drag reduction corresponds to the fully turbulent analyses, namely a reduction of the wing’s pressure drag, the increase in suction strength also acts on the pressure drag component for the reference configuration. Due to the limited extent of laminar flow, friction drag components remain nearly constant when sweeping over different suction coefficients and, therefore, the expected synergy effects are not directly observable for the CATeW-01 reference aircraft beyond the scope of LFC. This highlights the necessity of further investigations to draw in-depth conclusions with respect to the technologies’ coupling effects. The development of an optimized wing geometry for HLFC application is, therefore, foreseen within the project, allowing for comparisons with the LowFi-aerodynamic toolchain and achieving the key requirement of extended areas of laminar flow downstream of the suction panel.

## List of symbols

ADHF: Adaptive dropped hinge flap
CATeW: Coupled aerodynamic technologies for aircraft wings
CFD: Computational fluid dynamics
HiFi: High-fidelity
HLFC: Hybrid laminar flow control
LILI: DLR LIFTING$\_$LINE
LowFi: Low-fidelity
MICADO: Multidisciplinary integrated conceptual aircraft design and optimization
OAD: Overall aircraft design
RANS: Reynolds-averaged Navier–Stokes
ROM: Reduced order model
VC: Variable camber
$\alpha$: Angle of attack $\left[ \circ \right]$
$\eta$: Non-dimensional wing span [−]
$$\mathrm {Ma}$$: Mach number [−]
$$\mathrm {Re}$$: Reynolds number [−]
*c*: Chord length [*﻿m*]
$$C_D$$: Drag coefficient [−]
$c_f$: Skin friction coefficient [−]
$C_j$: Suction mass flow coefficient [−]
$$C_L$$: Lift coefficient [−]
$$c_p$$: Pressure coefficient [−]
$C_{D,f}$: Friction drag coefficient [−]
$C_{D,p}$: Pressure drag coefficient [−]
$$U_\infty$$: Freestream velocity [*m*/*s*]
$V_n$: Wall-normal velocity [*m*/*s*]
*x*, *y*, *z*: Body-fixed cartesian coordinates [*m*]
$$_\mathrm {cr}$$: Cruise
$$_\mathrm {H}$$: Hinge
$$_\mathrm {loc}$$: Local

## Footnotes

1 The LILI code has been developed at DLR [26, 27] and is often used in conceptual aircraft design, because, when compared to higher order methods such as Reynolds-averaged Navier–Stokes (RANS) computations, it gives reasonable results even for transonic flow conditions [28].

2 Drag decomposition performed in this paper is based on readily available results from surface integration of normal and tangential forces on viscous walls, leading to the typical split into drag forces stemming from pressure and friction components [48]
